# Investigating the physiology of normothermic ex vivo heart perfusion in an isolated slaughterhouse porcine model used for device testing and training

**DOI:** 10.1186/s12872-019-1242-9

**Published:** 2019-11-11

**Authors:** Benjamin Kappler, Carlos A. Ledezma, Sjoerd van Tuijl, Veronique Meijborg, Bastiaan J. Boukens, Bülent Ergin, P. J. Tan, Marco Stijnen, Can Ince, Vanessa Díaz-Zuccarini, Bas A. J. M. de Mol

**Affiliations:** 1 Department Cardiothoracic Surgery, Amsterdam University Medical Center, Meibergdreef 9, Amsterdam, The Netherlands; 2 LifeTec Group B.V, Eindhoven, The Netherlands; 3 Department of Mechanical Engineering, University College London, Torrington Place, London, UK; 4 Department of Medical Biology, Amsterdam University Medical Center, Meibergdreef 9, Amsterdam, The Netherlands; 5 Department of Translational Physiology, Amsterdam University Medical Center, Meibergdreef 9, Amsterdam, The Netherlands; 6 WEISS Centre for Surgical and Interventional Sciences, UCL, Gower Street 10, London, UK

**Keywords:** Biomarkers, Ex vivo, Normothermic perfusion, Cardiac physiology, Cardiac electrophysiology

## Abstract

**Background:**

The PhysioHeart™ is a mature acute platform, based isolated slaughterhouse hearts and able to validate cardiac devices and techniques in working mode. Despite perfusion, myocardial edema and time-dependent function degradation are reported. Therefore, monitoring several variables is necessary to identify which of these should be controlled to preserve the heart function. This study presents biochemical, electrophysiological and hemodynamic changes in the PhysioHeart™ to understand the pitfalls of ex vivo slaughterhouse heart hemoperfusion.

**Methods:**

Seven porcine hearts were harvested, arrested and revived using the PhysioHeart™. Cardiac output, SaO2, glucose and pH were maintained at physiological levels. Blood analyses were performed hourly and unipolar epicardial electrograms (UEG), pressures and flows were recorded to assess the physiological performance.

**Results:**

Normal cardiac performance was attained in terms of mean cardiac output (5.1 ± 1.7 l/min) and pressures but deteriorated over time. Across the experiments, homeostasis was maintained for 171.4 ± 54 min, osmolarity and blood electrolytes increased significantly between 10 and 80%, heart weight increased by 144 ± 41 g, free fatty acids (− 60%), glucose and lactate diminished, ammonia increased by 273 ± 76% and myocardial necrosis and UEG alterations appeared and aggravated. Progressively deteriorating electrophysiological and hemodynamic functions can be explained by reperfusion injury, waste product intoxication (i.e. hyperammonemia), lack of essential nutrients, ion imbalances and cardiac necrosis as a consequence of hepatological and nephrological plasma clearance absence.

**Conclusions:**

The PhysioHeart™ is an acute model, suitable for cardiac device and therapy assessment, which can precede conventional animal studies. However, observations indicate that ex vivo slaughterhouse hearts resemble cardiac physiology of deteriorating hearts in a multi-organ failure situation and signalize the need for plasma clearance during perfusion to attenuate time-dependent function degradation. The presented study therefore provides an in-dept understanding of the sources and reasons causing the cardiac function loss, as a first step for future effort to prolong cardiac perfusion in the PhysioHeart™. These findings could be also of potential interest for other cardiac platforms.

## Background

Isolated perfused hearts have been used for cardiac research since the groundbreaking work of Langendorff [1] in 1895. Hearts isolated from the body and perfused ex vivo offer results with higher reproducibility, when compared to in vivo counterparts, because they are not affected by systemic influences, such as neurohumoral control and systemic circulation. Extensive work has been done to customize ex vivo heart platforms for precise research purposes and to improve and accelerate the development of cardiac prototypes and interventions. The optimal perfusion with warm oxygenated blood enables realistic device validation, while these setups can be also medical devices themselves (e.g. donor heart transportation) [2, 3]. Nowadays, ex vivo models are available that offer more in-dept research possibilities such as electrophysiological studies [4, 5] and working heart studies [6, 7], in which blood is pumped in a natural way (i.e. the blood enters the heart through the left atrium, it is then pumped to the left ventricle and it is finally ejected through the aorta). This “working mode” allows measurements of pump function, cardiac pressures (i.e. ventricular, aortic, pulmonary) and flows (i.e. aortic, coronary, etc.) and was first described by Neely, Liebermeister [6] in 1967. As a result of these setup developments, isolated heart preparations are used for a variety of investigations in cardiology, cardiac surgery, physiology and pharmacology to investigate physiological, biochemical, pharmacological and morphological characteristics as well as cardiac function [8–13].

Pig hearts are appreciated for investigations, which specifically require physiological conditions similar to patient application, as these hearts are a good match to the morphology and physiology of human hearts [10]. However, potential significant differences (i.e. shape, opening of superior and inferior caval veins into the atrium, prominent left azygous vein drainage, number of pulmonary veins, etc.) are known between porcine and human hearts [14].

Considering the increase of cardiac investigations while bearing in mind the costs and ethical issues related to laboratory animal experiments, isolated slaughterhouse hearts could under carefully chosen circumstances be used for feasibility studies that can precede the conventional and contentious animal testing. Slaughterhouse heart experiments are less cost intense, as experimentation protocols do not need ethical approval, while uncertified teams can perform several experiments a day, due to the abundance of slaughterhouse hearts without sacrificing additional animals for the research conducted. This results in an improved learning curve as investigations can originate faster [15].

One customized model based on slaughterhouse hearts is the PhysioHeart™, developed by LifeTec Group B.V. (Eindhoven, The Netherlands). Slaughterhouse pig hearts revived in this commercially available isolated heart model have previously shown cardiac output, stroke volume, pressures, valve interactions and dynamic changes that are comparable to those observed in humans [15]. In the last decade, this model has been used to successfully visualize transcatheter aortic valve implantations [16], to assess computer tomographic myocardial perfusions [17], to evaluate magnetic resonance imaging-based 4D flow analysis and to study left ventricular assist devices [18, 19], intra-aortic balloon pump support [20] and coronary autoregulation [21].

In the face of, extensive work, novelties and decades of experience, isolated heart perfusion remains demanding, in particular in use of slaughterhouse hearts. The unavoidable warm ischemia (between exsanguination of the pig and the cardiac arrest) and the cold transport to the laboratory influence the experimentation outcome negatively. These shortcomings encountered within these experiments, specifically the time-dependent contractility degradation and edema, are under specific aspects similar to those observed within human DCD (donation after circulatory determined death) heart preservation, which are considered for transplantations [22].

Although the PhysioHeart™ model is well-established for device and therapy testing, less is known about its cardiac metabolic, biochemical and electrical physiology as the experiments progress in time, specifically regarding the source of the abovementioned shortcomings. Therefore, in this study, we report a comprehensive recording of time-dependent metabolic, biochemical, electrical and hemodynamic variables acquired from isolated normothermic, hemoperfused, slaughterhouse porcine hearts. The goal of this study was to create a much as possible complete inventory of the changes over time to identify the causes of the progressive deterioration of cardiac function and development of edema in the PhysioHeart™. This study provides the basis for further investigations and improvements to extend the cardiac function of the slaughterhouse hearts revived in the PhysioHeart™ platform. We envision that this study with its comprehensive recordings could be of potential interest for cardiac normothermic perfusion in other platforms.

## Methods

### Animals

Seven hearts were obtained from Dutch Landrace pigs slaughtered for human consumption. Each animal had a weight of approximately 110 Kg. All protocols followed by the slaughterhouse and laboratory were consistent with EC regulations 1069/2009 regarding the use of slaughterhouse animal material for diagnosis and research, supervised by the Dutch Government (Dutch Ministry of Agriculture, Nature and Food Quality) and were approved by the associated legal authorities of animal welfare (Food and Consumer Product Safety Authority).

### Isolation and administration of cardioplegia

The procedure for harvesting the hearts was equivalent in all the animals and is summarized in this section. Before heart harvesting, the pig was electrically stunned, hung and exsanguinated, but not heparinized. Afterwards a parasternal incision was made in the thorax and the heart and lungs were removed *en-bloc*. The heart was immediately topologically cooled. Subsequently, the pericardial sac was opened, the pulmonary artery was transected under the bifurcation and the aorta was transected under the first supra-aortic vessel. The heart was then isolated and prepared as described in a previous study [21]. Immediately after removal, the aorta was cannulated and 2 L of heparinized modified St. Thomas 2 crystalloid cardioplegic solution (Table 1) was administered through the coronary arteries at a mean pressure of 80–100 mmHg and a temperature of 4 °C. Warm ischemic time was measured and never exceeded 4 minutes. During the harvesting, 10 L blood to be used for reperfusion were collected, by exsanguination, from subsequently slaughtered pigs; this blood was also heparinized at 5000 IU/L. The heart was transported to the laboratory submerged in the St. Thomas solution at 4 °C and the blood was transported in a Jerry can at room temperature with no additional treatments. The heart and blood were stored for 2 hours (transport and preparation) and, after 1 hour in storage, an additional liter of cold cardioplegic solution was administered to the heart and the blood was filtered with a 200 μm filter.

**Table 1:** Modified St. Thomas solution for crystalloid cardioplegia

Salts	Concentrations (mmol)
Sodium	130
Potassium	16
Magnesium	16
Calcium	1.2
Chloride	171.4
Procaine	1
Heparin	5000 IU/L

### Mounting isolated hearts onto the circuit

**Fig. 1:**
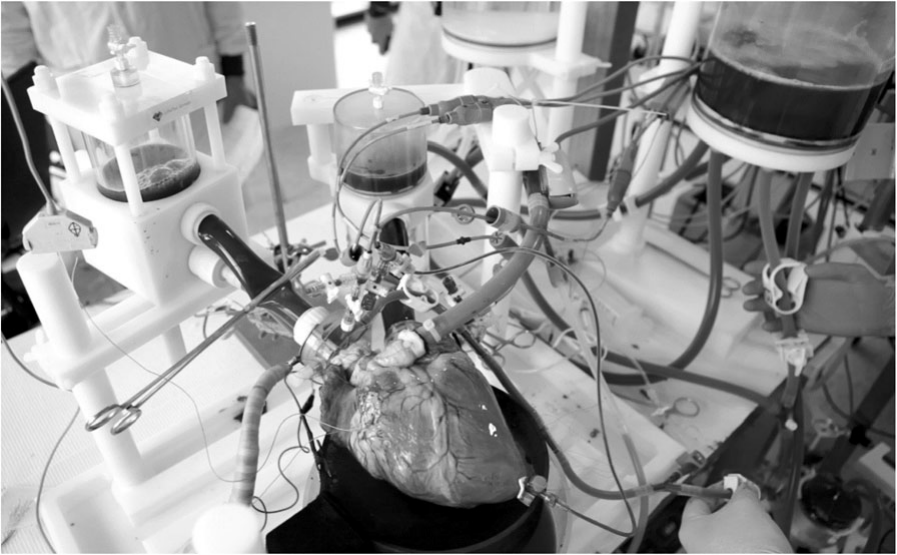
Porcine heart mounted in the PhysioHeart™ platform

The ex-vivo perfusion of the slaughterhouse hearts was performed using the PhysioHeart™ platform, which has been described previously [15]. The left atrium and the aorta were connected to the platform, the pulmonary artery was cannulated in order to return the venous blood to the reservoir and measure coronary flow. Temporary pacing leads (Medtronic Inc., Minneapolis, MN, USA) were placed on the right ventricular outflow tract (RVOT) to monitor electrical activity and paced when needed to maintain a regular rhythm. A porcine heart mounted in the platform in shown in Fig. 1. The perfusion circuit was primed with normothermic (38 °C) heparinized oxygenated blood (6 L, hematocrit 18–25%; pH 7.40 ± 0.05) and supplemented with insulin (0.32 units/L). The hearts were then perfused in Langendorff mode to keep the coronary perfusion pressure at 80 mmHg. Steady contractile myocardial activity was restored within 5 minutes, providing defibrillation when needed.

**Fig. 2:**
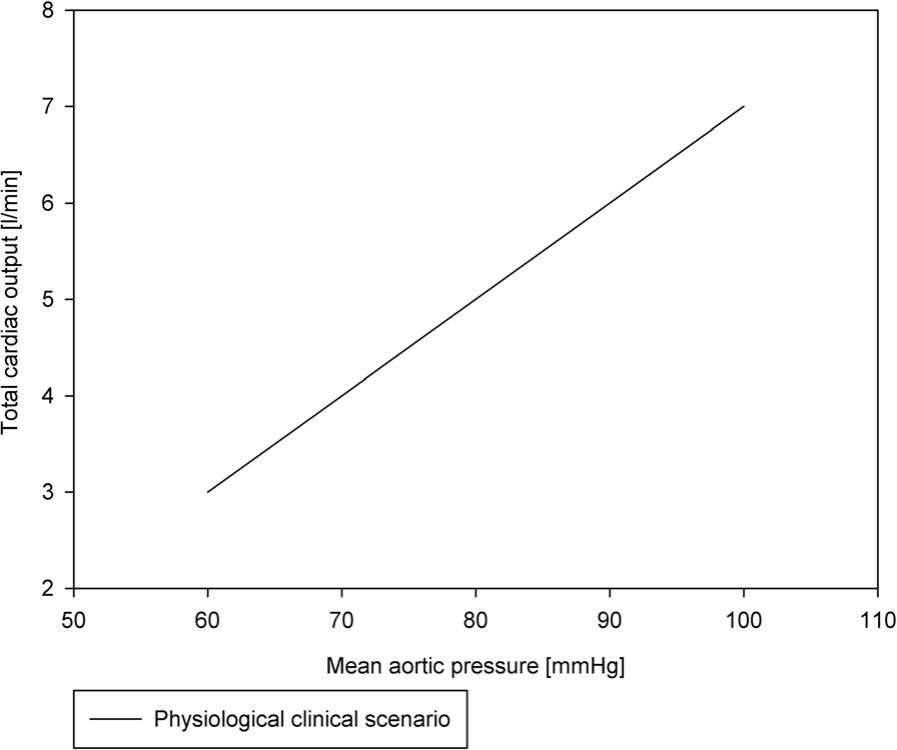
Physiological clinical scenario followed during the PhysioHeart™ experiments

After a supplementary stabilization time of about 15 min, preload and afterload were opened, so the platform was switched from Langendorff to working mode. During the working mode, the left ventricle (LV) ejects the perfusate into the afterload. The atrial pressure (ATP) and aortic pressure (AP) were adjusted to create a mean load of 10–20 mmHg and of 60–100 mmHg, respectively. All related pressures and flows were monitored and kept at physiological values. The blood glucose level was maintained, manually, between 5 and 7 mmol/L by the addition of a mixture of glucose and insulin. The pH was maintained with sodium bicarbonate. The mean cardiac output and coronary flow rate were measured using two ultrasound flow probes (SonoTT™ Clamp-On Transducer, em-tec GmbH, Finning, Germany), placed after the afterload and the pulmonary artery, respectively. The hemodynamic parameters were continuously monitored and adapted according to the pump function of the heart to fit the optimal clinical scenario as reported in Schampaert, van Nunen [20], shown in Fig. 2.

### Blood analysis and control

Arterial blood samples were taken from the oxygenator before the heart was connected to the loop, this was called the baseline measurement, and then every 60 min after reperfusion. Blood gas values, temperature, and electrolytes were measured using a VetScan i-STAT 1 (Abaxis, Union City, CA, USA). Based on the i-STAT 1 measurements, the pH, glucose, and ionized calcium were maintained at physiological levels by adding sodium bicarbonate, a glucose/insulin mixture (2 mmol/U) and calcium chloride. For further detailed analysis, full blood was collected in blood collection tubes. Tubes for plasma and serum analysis were centrifuged at 500 g for 10 min. Plasma and full blood samples were stored at 4 °C and serum samples were stored at − 80 °C overnight. The following day, samples were transported for analysis to a clinical laboratory (Máxima Medisch Centrum, Veldhoven, the Netherlands) and were examined with a C8000 analyser (Roche).

### Pacing protocols, electrical acquisitions and signal processing and analysis

**Fig. 3:**
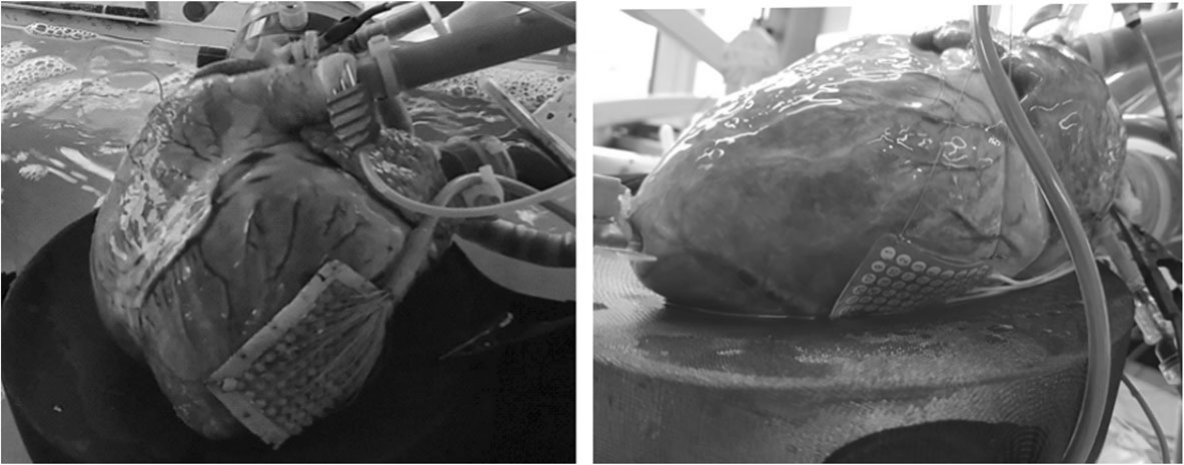
Electrode grids used to measure the UEG during the PhysioHeart™ experiments. (left) 11 × 11 electrode grid used with BioSemi ActiveTwo acquisition system (right) 6 × 8 electrode grid used with the National Instruments acquisition system

After the first four experiments, it was hypothesized that the analysis of the hearts’ electrophysiology could give further insight on how to prolong normothermic perfusion. Hence, as a proof of feasibility, an electrophysiological analysis was performed in the last three experiments as follows. The hearts were paced, when necessary, with a pacing lead placed at the RVOT. To avoid breakthrough beats, pacing was provided at a higher frequency than the exhibited sinus rhythm of around 100 bpm. In order to investigate their electrical restitution (i.e. the physiological change in wave propagation velocity with respect to the change in heart rate), the hearts were paced at 100, 120 and 150 bpm during working mode. Unipolar epicardial electrograms (UEG) were acquired, on the left ventricle, with two different custom-made acquisition systems. Using two different systems allowed to compare which would be a better option in cardiac transplant applications. First, the UEG were measured using a square grid containing 11 × 11 electrodes, with 5 mm inter-electrode spacing. When using this grid, the UEG were recorded, simultaneously from all electrodes, using a BioSemi ActiveTwo acquisition and preprocessing system at a sampling frequency of 2048 Hz. The second acquisition system was formed by a rectangular grid of 6 × 8 electrodes (AD-TECH FG48G-SP05X-0E2). The signals from the latter grid were recorded simultaneously from all electrodes by a National Instruments (NI) 6031E acquisition card at 500 Hz. The acquisition card was connected to the grid using an NI SCB-100 shielded connector block followed by a NI-SH100100 connector cable. The configuration of the acquisition card and the recording of the signals was made using a custom-made virtual instrument developed in NI Labview 2013. Both grids are shown, as placed during the experiments, in Fig. 3.

After their acquisition, the UEG were pre-processed using a digital, Butterworth, band-pass filter with cutoff frequencies *fc*_1_ = 0.5 Hz and *fc*_2_ = 40 Hz. This filter removed undesirable DC components, baseline wander and high frequency, low-amplitude, noise. Afterwards, the activation time of the tissue under each electrode was measured as the point when the signal’s derivative was minimal during the QRS complex of each beat. The propagation of the activation wave was assessed using activation maps, these were constructed, for each beat, by measuring the activation time of the tissue under each electrode and drawing isochrones that joined the areas of the LV that activated simultaneously. Finally, the velocity at which the activation wave propagated, called wave propagation velocity (WPV), was measured from the activation maps using the following equation:

$$ WPV=\frac{d\left({p}_2,{p}_1\right)}{AT\left({p}_2\right)- AT\left({p}_1\right)} $$

where *p*_2_ and *p*_1_ are two points in the direction of propagation, *d*(*p*_2_, *p*_1_) is the Euclidean distance between the two points and *AT*(*p*) is the activation time at point *p*. The UEG post-processing was done using Matlab (R2017b).

### Statistics

The seven hearts selected for this study were those that showed initial physiological cardiac hemodynamics which we considered to be at least CO 3 L/min, ATP 10–20 mmHg and AP 60 mmHg at the beginning of the working mode. Statistically significant differences in the mean values among treatment groups were determined by one-way analysis of variance. A paired t-test was used to confirm differences between heart weights before and after the experiment. A *p*-value ≤0.05 was set as a criterion for significance. All values are presented as the mean ± standard deviation. All the statistical tests were performed with Sigmaplot 11.0.

## Results

### Cardiac hemodynamics and weight

**Fig. 4:**
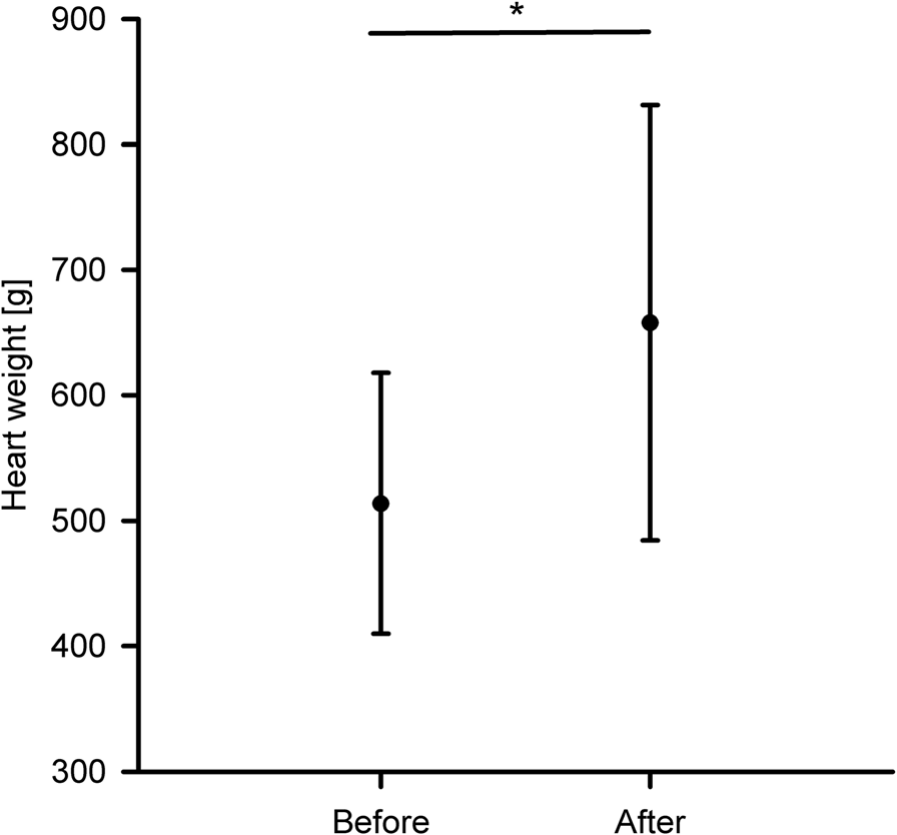
Weight of the hearts before connecting them to the platform and after the end of the experiments, * indicates a statistically significant difference

The porcine hearts used in the experiments had a mean weight of 513 ± 104 g and showed hypertrophic cardiomyopathy before they were connected to the experimental platform, a general observation on the use of slaughterhouse hearts. After the experiments, the hearts had increased their weight to 657 ± 173 g, which is a statistically significant (*p* = 0.018) increase of 28%. This result can be observed in Fig. 4.

**Fig. 5:**
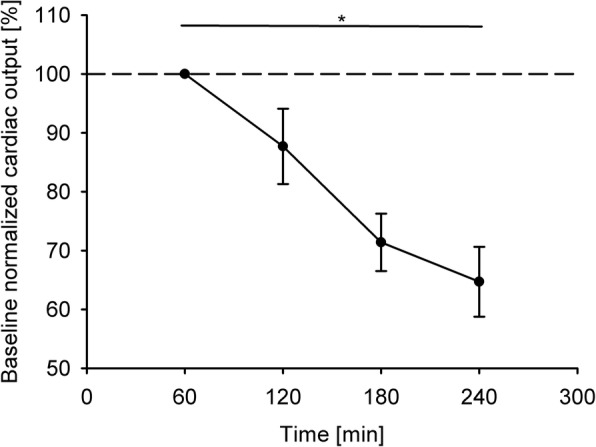
Progression of the cardiac output across all the experiments. The values are presented as a percentage of their baseline value. * indicates a statistically significant difference

All seven hearts were revived and produced initial normal cardiac flows under the relevant pressures (ATP, 13 ± 2 mmHg; LVP, 71 ± 18 mmHg; AP,75 ± 8 mmHg) during the working mode. At baseline, the initial cardiac output (5.1 ± 1.7 L/min) was physiological in all hearts but decreased over time with a deterioration rate of 12.5 ± 2.7% per hour, while pressures were kept at physiological levels. All hearts preserved physiological hemodynamics for at least 2 hours. Only two of the seven hearts were able to maintain physiological hemodynamics for up to 4 hours. The progression of the cardiac output during the experiments can be observed in Fig. 5; in the figure, the values are shown as a percentage of their baseline values. The figure indicates that the degradation of the cardiac output was similar across all the experiments. The coronary flows and aortic pressures degraded with a similar trend.

### Blood values

#### Electrolytes

**Fig. 6:**
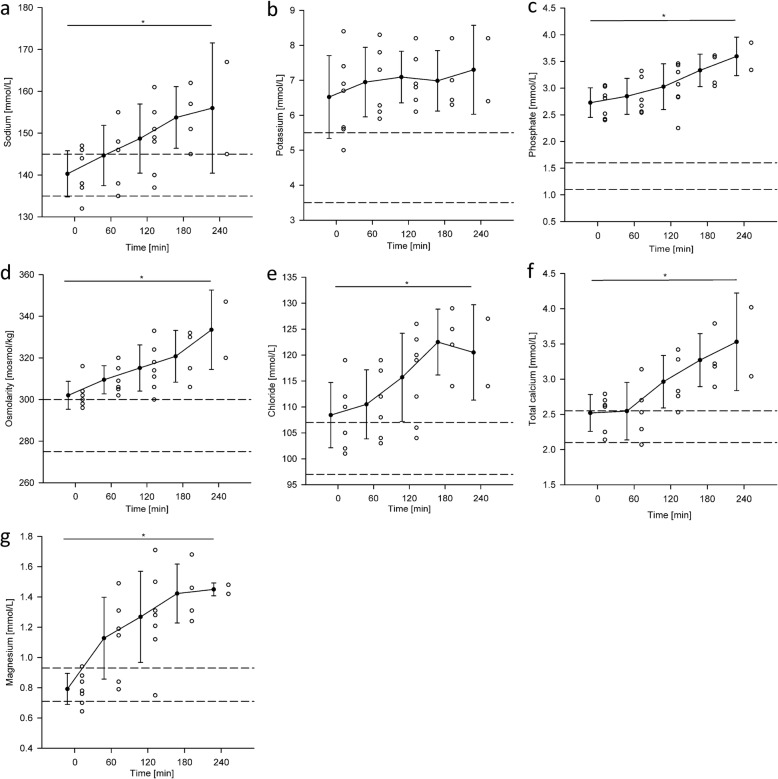
Time dependent changes in electrolyte concentrations shown as scatter and column plots. The dashed lines indicate upper and lower reference limit. **a**. Sodium, **b**. Potassium, **c**. Phosphate, **d**. Osmolarity, **e**. Chloride, **f**. Total calcium, **g**. Magnesium. * denotes a statistically significant difference in mean value

The electrolyte concentrations that were documented across all seven experiments are presented in Fig. 6. At baseline, before connecting the hearts into the circuit, hyper-chloremia, −kalemia, −phosphatemia and -osmolarity were observed. Potassium values remained at its baseline level (7 ± 0.3 mmol/L) during the experiments. Sodium, total calcium, chloride, phosphate and magnesium increased steadily each hour by 3.9 ± 1.2 mmol/L, 0.3 ± 0.2 mmol/L, 4.7 ± 2.4 mmol/L, 0.2 ± 0.08 mmol/L and 0.2 ± 0.1 mol/L respectively, which resulted in hyper-natremia, −calcemia and -magnesemia during reperfusion. In all experiments, the electrolyte concentrations in blood were unphysiologically high before reperfusion or rose to unphysiological concentrations as the experiment progressed.

#### Metabolic panel

**Fig. 7:**
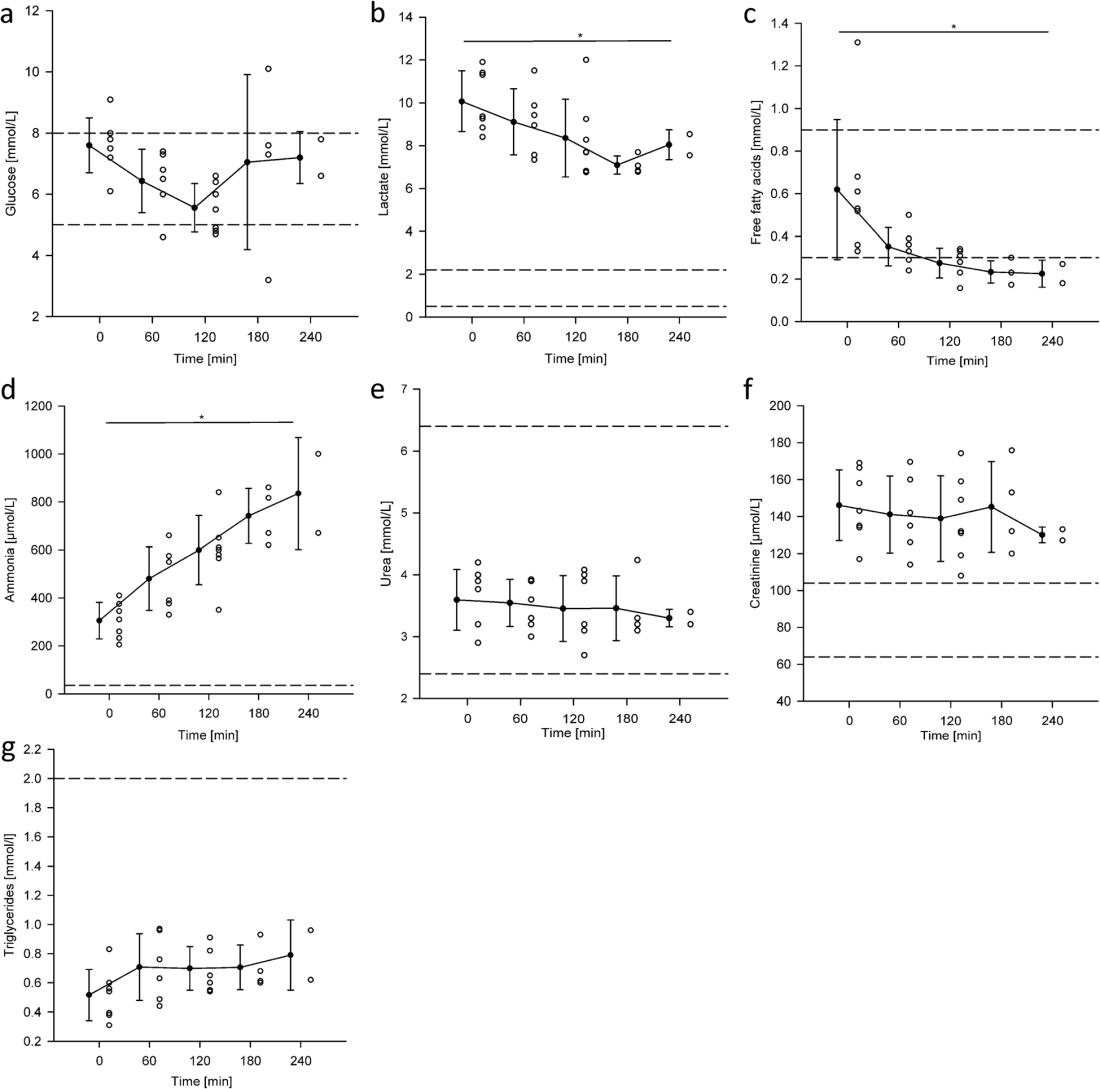
Scatter and column plots showing the changes of metabolic biomarker concentrations during the ex-vivo beating heart experiments. Dashed lines indicate upper and lower reference limits. **a**. Glucose, **b**. Lactate, **c**. Free fatty acids, **d**. Ammonia, **e**. Urea, **f**. Creatinine, **g**. Triglycerides. * denotes a statistically significant difference in mean value

The metabolic panel, and its evolution in time, is presented in Fig. 7. The hearts revived in the platform showed symptoms of an aerobic metabolism during the working mode. As can be observed in Fig. 7b, this was evidenced by the consumption of approximately 1 mmol/L of glucose and 1 mmol/L of lactate per hour. However, lactate levels stabilized or even rose in the last hour, this is an indication of an anaerobic metabolism towards the end of the experiment. The manual adjustments in glucose helped maintaining it within physiological ranges but resulted in a wide variation during the third hour with an increase in the mean value, as can be observed in Fig. 7a. Additionally, as can be observed in Fig. 7c the mean values of free fatty acids decreased with an exponential trend from 0.62 ± 0.33 mmol/L to 0.22 ± 0.06 mmol/L in the first hour and dropped below the lower reference limit in the second hour; this is also an indication of an aerobic metabolism. Concentrations of triglycerides were stable at 0.7 ± 0.1 mmol/L during the experiment; however, an increase in the mean value after 1 hour of reperfusion can be observed in Fig. 7g. Urea and creatinine were stable at 3.5 ± 0.1 mmol/L and 140.2 ± 6.4 μmol/L respectively with signs of elevated creatinine levels. In turn, hyperammonemia could already be observed at baseline (305 ± 76 μmol/L) and increased at a rate of 132.5 ± 34.2 μmol/L per hour during the experiment.

#### Cell damage biomarkers

**Fig. 8:**
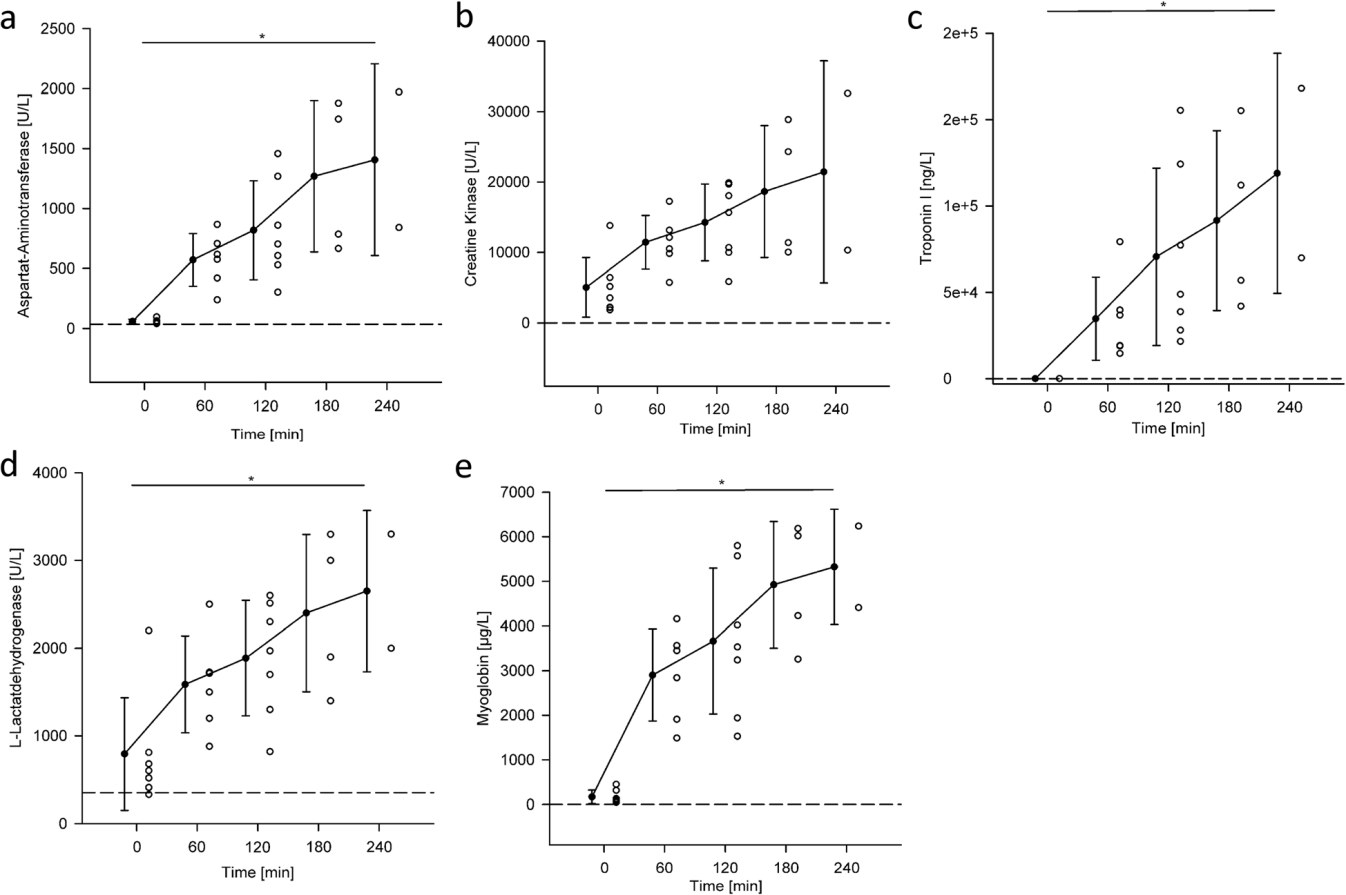
Scatter and column plots showing the cardiac necrosis markers values during hemoperfusion in the PhysioHeart™ platform. **a**. Aspartat-Aminotransferase (ASAT), **b**. Creatine Kinase (CK), **c**. Troponin I, **d**. L-Lactatdehydrogenase (LDH) and **e**. Myoglobin. * denotes a statistically significant difference mean value

Before connecting the hearts to the circuit, the mean values of the cell damage markers were above the reference limits, as can be observed in Fig. 8. Within the first hour after cardiac reperfusion, all cell damage markers showed the highest increase compared to the later measurements. Aspartat-Aminotransferase (ASAT), Creatine Kinase (CK), Troponin I, L-Lactatdehydrogenase (LDH) and Myoglobin were rising throughout the experiment with 337 ± 175 U/L, 4099 ± 1699 U/L, 29717 ± 6954 ng/L, 464 ± 1699 U/L, 1289 ± 1026 μg/L respectively.

#### Additional plasma values

**Fig. 9:**
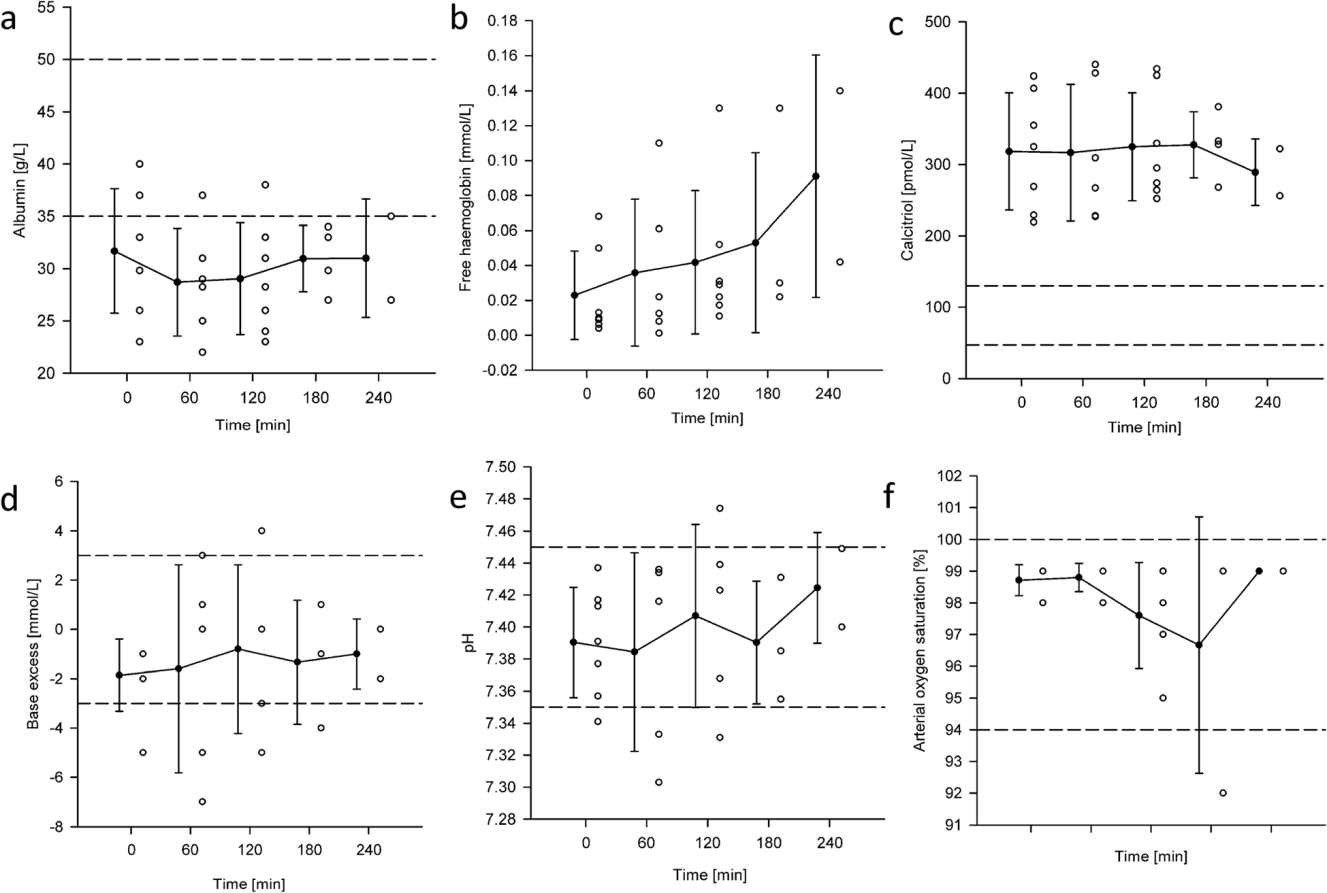
Scatter and column plots of blood related biomarkers during PhysioHeart™ experiments. **a**. Albumin, **b**. Free hemoglobin, **c**. Calcitriol, **d**. Base Excess, **e**. pH, **f**. Arterial oxygen saturation (SaO2). * denotes a statistically significant difference in mean values

Manual pH balancing ensured the stability of the mean values of pH (7.40 ± 0.02) and base excess (− 1.32 ± 0.43 mm/L) but resulted in high variance in these markers, see Fig. 9d and e. Although albumin concentrations (28 ± 3 g/L) were also stable, their levels were low in relation to the reference range and identified the pathological nature of hypoalbuminemia. Similarly, hypervitaminosis D was identified due to high and not changing calcitriol levels (315 ± 15 pmol/L) above the reference range, see Fig. 9c. Finally, free hemoglobin increased during the experiment by approximately 0.02 ± 0.01 mmol/L per hour.

### Activation maps and wave propagation velocity measurements

**Fig. 10:**
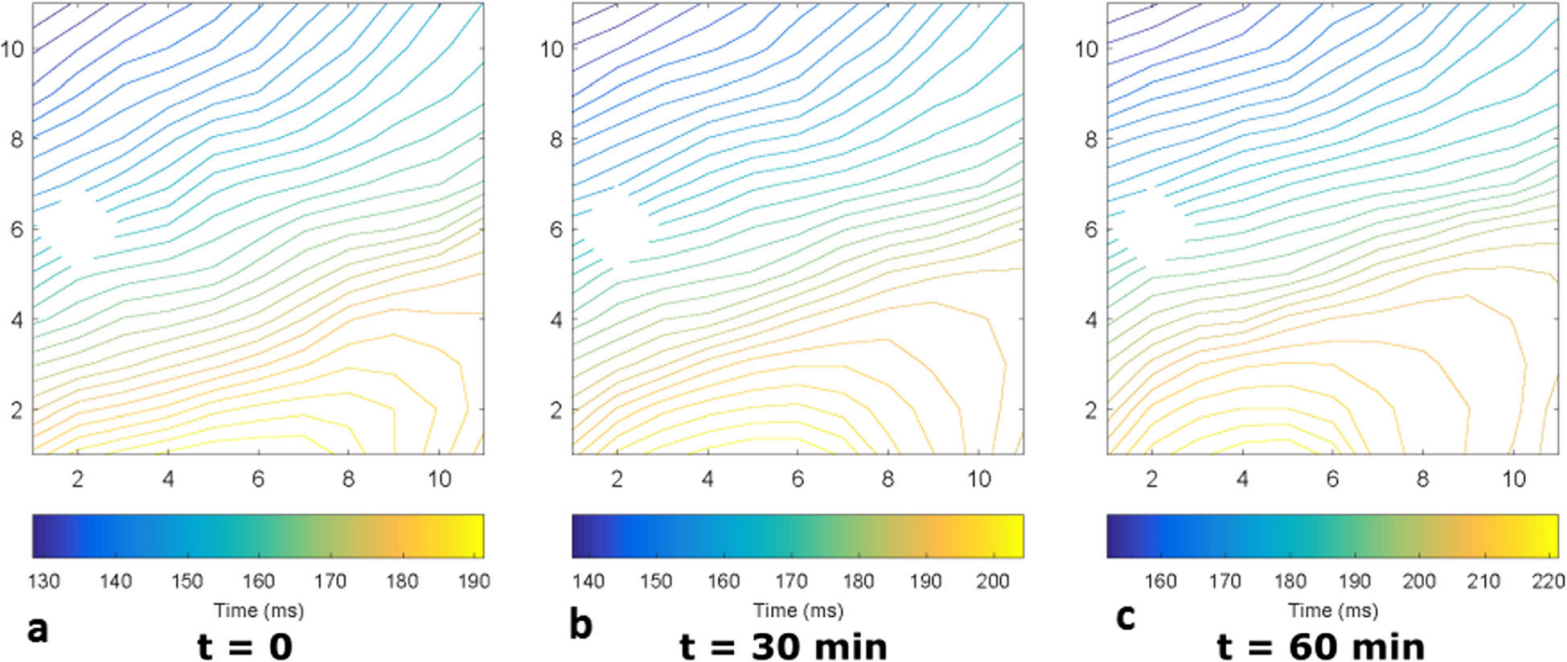
Activation maps observed during the working mode of the baseline PhysioHeart™ experiment. The heart was paced at 100 bpm. Electrode (2,6) malfunctioned, so the data from that channel was ignored. **a**. Was measured at the beginning of the working mode, **b** was measured 30 min after **a**. and **c**. was made 30 min after **b**. at the end-point of the experiment

**Fig. 11:**
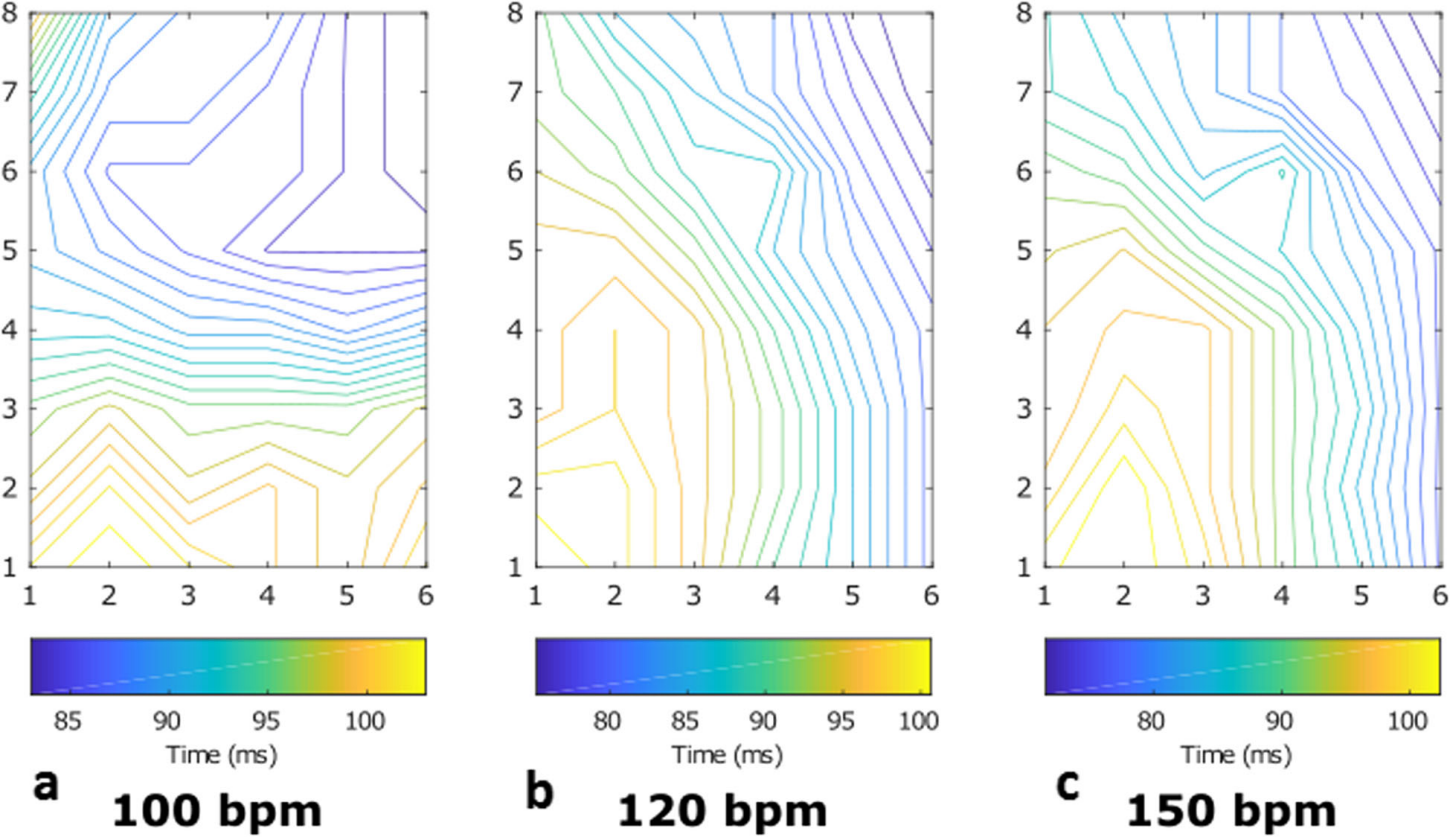
Activation maps observed during the working mode of a PhysioHeart™ at three frequencies. Three different frequencies (sinus rhythm at 100 bpm, 120 bpm and 150 bpm) have been included in the measurement. In **a**. the heart was beating at sinus rhythm, whereas in **b**. and **c**. a pacemaker drove the beating of the heart

Activation maps have been recorded on the epicardial surface of the hearts with two different electrode grids (11 × 11: Fig. 10, 6x8: Fig. 11). Figure 10 shows representative examples, at three different instants in time, of the activation maps obtained during one of the experiments, in which the heart was paced at 100 bpm during working mode. The activation patterns do not show signs of conduction block or arrhythmic nodes at any moment of the working mode on the area covered by the grid. However, the three activation patterns show a delay in the arrival of the depolarizing wave over time. The moment the wave arrives to the grid in Fig 10 Fig 11a is at around 130 ms after the pacing signal, this is increased to around 140 ms in Fig. 10b and is over 150 ms at the endpoint of the experiment (see Fig. 10c).

The activation maps measured in another experiment included a pacing protocol, in which three different frequencies (at sinus rhythm at 100 bpm, 120 bpm and 150 bpm) have been considered, which can be observed in Fig. 11. These measurements were made using the 6 × 8 electrode grid configuration. The direction of the depolarizing wave during pacing (Fig. 11b-c) was different compared to sinus rhythm (see Fig. 11a), since the natural heart rhythm starts in the sinus node and the pacing is provided at the RVOT, this phenomenon is justified. As before, the region that was monitored by the grid showed no arrhythmic nodes or areas of conduction block during the working mode. Furthermore, as can be observed in Fig. 11b-c changing pacing frequencies had no observable effect on the propagation pattern.

Differences between the activation maps in Fig. 10 and 11 are a consequence of the grid configurations. Activation maps in Fig. 10a-c look more uniform due to the larger grid (11 × 11 electrodes, 55x55mm) compared to Fig. 11a-c (6 × 8 electrodes, 30x48mm), which is also reflected in the shorter travelling time in Fig. 11. Differences in direction are attributable to the different positions of the grids (see Fig. 3).

The wave propagation velocities measured across all PhysioHeart™ experiments at the beginning of the working mode are presented in Table 2, they indicate normal physiological behavior. Indeed, the mean value of the wave propagation velocity is within the boundaries observed in healthy hearts [23] and it exhibits a physiological restitution effect [24], (i.e. a decrease in both action potential duration and wave propagation velocity as the stimulation frequency increases). However, the standard deviation of the WPV shows a beat-to-beat variability, of around 10 cm/s, which is unphysiological when compared to the relatively small variability observed in healthy measurements [23]. Also, towards the end of the experiment, the measurements of wave propagation velocity indicated a value of around 65 cm/s at 100 bpm (not shown in the table). This decrease in velocity indicates an impairment of the electrical conduction as the experiment progresses.

**Table 2:** Mean value (μ_WPV_) and standard deviation (σ_WPV_) of the wave propagation velocities measured during working mode at different pacing frequencies. The table summarizes the acquisitions made across all PhysioHeart™ experiments

Pacing frequency	μ_WPV_	σ_WPV_
100 bpm	100.00 cm/s	9.10 cm/s
120 bpm	79.86 cm/s	13.31 cm/s
150 bpm	75.33 cm/s	9.92 cm/s

## Discussion

**Fig. 12:**
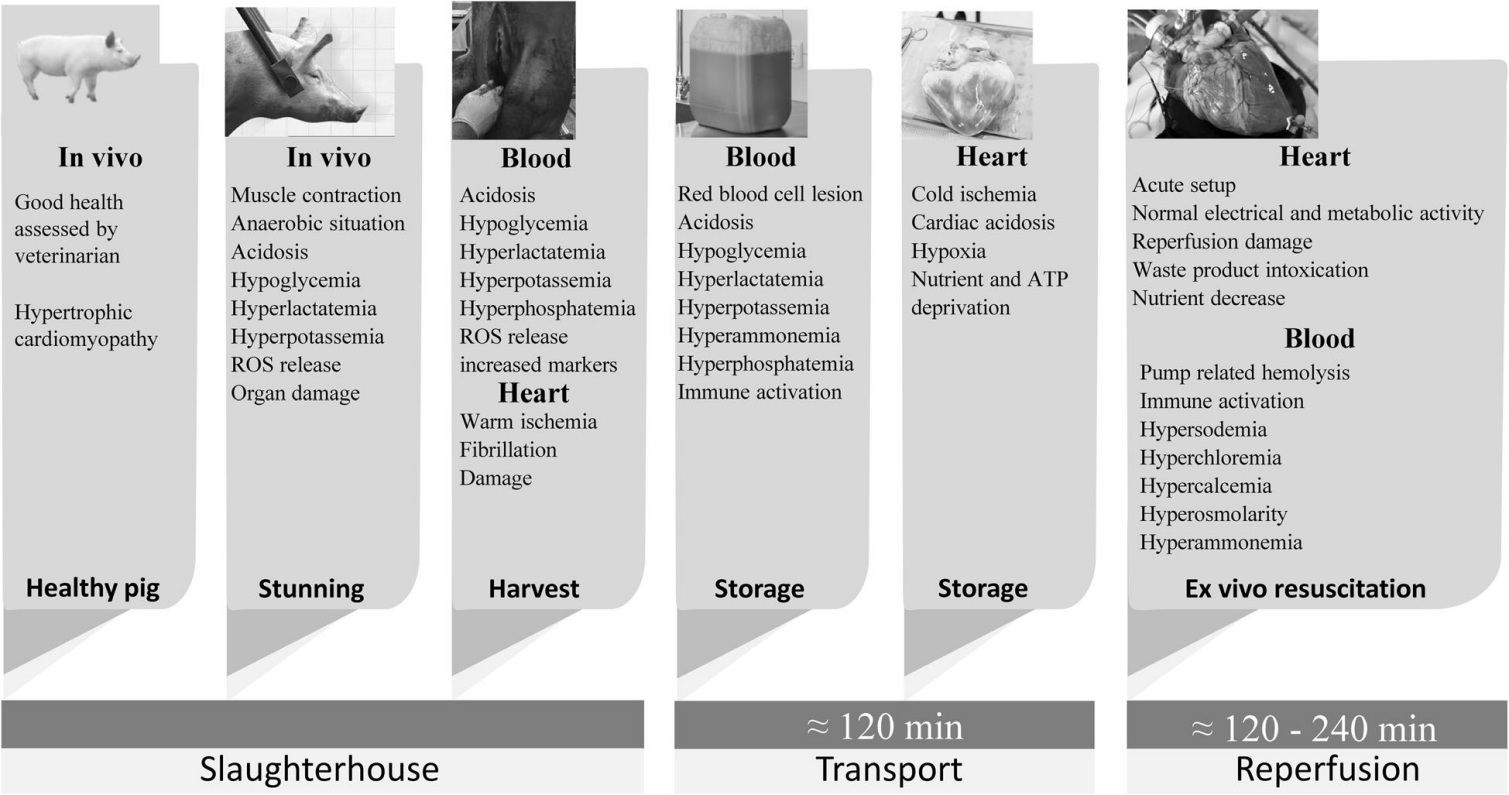
Timeline of process-dependent physiological changes during PhysioHeart™ experiments

The main objective of this study was to present an in-depth biochemical, hemodynamic and electrophysiological characterization of our isolated ex-vivo slaughterhouse heart experiments in the PhysioHeart™ platform. Initially, the resuscitated porcine hearts showed physiological metabolic, electrical and hemodynamic activities. However, electrophysiological and hemodynamic cardiac functions gradually diminished due to the initiation of waste product intoxication, reduction of essential nutrients, ion imbalances, cardiac necrosis and, most likely lastly, reperfusion injury and inflammation. On one hand, we conclude that the variability observed in the baseline pump function is a consequence of the ‘random’ selection of the slaughterhouse animals and the harvesting techniques. On the other hand, the superimposed progressive diminishment in cardiac function is concluded to be a result of the isolated slaughterhouse heart pathophysiology. Namely, the observed loss of function is associated with an increased level of metabolites and electrolytes, declining nutrients, a gradual loss of tissue integrity with edema and cell death which we believe is a result of the lack of hepatic and nephrological plasma clearance in the isolated heart setting. Figure 12 and Table 3 summarize these results, which resemble the deterioration of the heart function in a multi-organ failure situation. These observations support the use of plasma clearance interventions and support the working hypothesis that isolated hearts should be treated, as far as possible, as heart-and-organ failure environment.

**Table 3:** Result summary

Figure	Interpretation
Figure 4	− Hypertrophic cardiomyopathy, a general observation in the use of slaughterhouse hearts (before) − Swelling of the cardiac tissue during reperfusion (after)
Figure 5	− Diminishing cardiac functions, possibly due to the initiation of waste product intoxication, reduction of essential nutrients, ion imbalances, cardiac necrosis and, most likely lastly, reperfusion injury and inflammation
Figure 6	− Hyperpotassemia (b), hypermagnesemia (g) and hyperphosphatemia(c) due to cardiac necrosis and washout of cardioplegia − Hypersodemia (a) due to sodium bicarbonate administration (pH balancing), while calcium chloride was administered to counterbalance the calcium reduction caused by bicarbonate, resulting in hypercalcemia (f) and hyperchloremia (e) − Hyperosmolarity (d) due to increase of ions
Figure 7	− General physiological aerobic cardiac metabolism, supported by free fatty acid (c) uptake and lactate (b) as well as glucose (a) metabolism − Amino acid catabolism, confirmed by rise of ammonia (d) − Decrease of essential cardiac nutrients (e.g. free fatty acids) and increase of toxic waste products (e.g. ammonia) over time − Rise of lactate after 180 min signalizes ischemia and acidosis − Hyperuremia (e) − Hypercreatininemia (f) − Static triglycerides (g) verifies the exclusion of fluid evaporation as a cause for the ion increase
Figure 8	− Hypoalbumia (a) due to blood dilution and potential cause for edema − Slight elevation of free hemoglobin (b) caused by the centrifugal pump − Hypercalcitriolemia (c) − Inconspicuous pH (e) and base excess(d) values and atrial oxygenation (f)
Figure 9	− Severe cardiac necrosis possibly due to reperfusion injury and inflammation and, heterogeneous cardioplegia delivery in the slaughterhouse
Figures 10 & 11	− Physiological electrical activities of hearts during working mode − Unaltered electrical conduction pathways

The limited duration of acceptable performance during the isolated heart experiments highlights that the isolated working heart needs to be in an environment that resembles the in-vivo physiology to avoid loss of its morphological and functional integrity. The slaughterhouse pigs used in this study were in general good health and were examined by a veterinarian prior to slaughtering. However, previous research has shown that domestication, selective breeding, scarce physical activity and improved feeding efficiency lead to morphological abnormalities in slaughterhouse-derived porcine hearts [25]; this was observed in our specimens as hypertrophic cardiomyopathy. Also, the baseline blood measurements revealed elevated levels in damage markers (i.e. CK, ASAT, LDH, troponin and myoglobin). As previous research has shown, elevated damage markers were most likely caused by the limited heart capacity observed in farm animals due to an intensive selection pressure and high stress during regrouping, transport and slaughtering [26]. The baseline measurements also revealed high ion concentrations and hyperosmolarity in blood; as previously reported by Heinze and Mitchell [27], this was probably a consequence of water accumulation in the intra- and inter-cellular space caused by the electrical stunning. Moreover, it is believed that the hyperkalemia observed at baseline was a consequence of the rapid drop in pH produced by the slaughtering; this drop in pH is known to lead to a cellular intake of H^+^ and release of K^+^ as a physiological process of pH balancing [28]. Electrical stunning also produces muscle contraction, which leads to hypoglycemia, hyperlactatemia, elevated creatinine levels and hyperammonemia [29]. These contractions can further lead to acidosis (low pH) and hyperlactatemia, an effect that has previously been reported during epileptic seizures when the muscles suffer from hypoxia [30, 31].

For each heart experiment, blood from different pigs was collected immediately after exsanguination and stored for about 2 h until preparation for reperfusion. Generally, pooling blood leads to transfusion reaction in humans, but the particular characteristics of the porcine hematopoietic system make porcine blood pooling less harmful as it causes transfusion reaction only in very rare cases [32]. However, the storage lesion of erythrocytes, during which glucose is consumed, levels of 2,3-diphosphoglycerate (DPG) and ATP decrease, and ammonia and potassium levels increase [33–35], is most likely contributing to the pathological blood values observed already at baseline.

Despite the quick harvesting process, warm cardiac ischemia is still expected to occur and to cause cardiac nutrient deficiency, hypoxia, acidosis and necrosis. It is expected that these processes will continue to damage the tissue during the cold storage period. Finally, these already stressed, hypertrophic hearts, were stored in a St. Thomas solution 2, a hypooncotic solution that promotes the influx of water through the endothelial layer into the intracellular space; this causes a further risk of cardiac edema [36, 37]. A more complex composed cardioplegic solutions like Custodiol [38], Somah [39], Celsior [40, 41] or UWS [41], could be of favor during hypothermic storage of slaughterhouse hearts. However, the use of a more complex solution also requires a careful consideration of price and advantages, which are currently under evaluation.

Therefore, the here above described ‘slaughterhouse-associated’ adverse effects should not be ignored when comparing the results with the carefully removed heart. These effects result in an increased chance for a reduced preservation, loss of cardiac tissue and function of the slaughterhouse porcine hearts. Despites these limitations one can learn from this pig heart the following:

### Biomarkers and electrolytes

Immediately after cardiac resuscitation, an increase in potassium and magnesium in blood is observed. This is probably due to the washout of the cardioplegic solution from the coronary system. This solution, which is administered during harvesting, contains potassium and magnesium at 16 mmol/L to ensure cardiac arrest during storage. Figure 6g illustrates this wash out on the example of magnesium which experiences its largest increase in the first hour.

Throughout the experiment, we observe a rise in cardiac injury markers caused by reperfusion injury [42] and inflammatory responses of leukocytes and platelets. It remains speculative, but possible causes for the increasing markers may be heterogeneous cardioplegia delivery to the myocardium, harvest-related thrombosis, air emboli, and/or hypertrophic myocardium. These circumstances vary amongst hearts and therefore result in the observed fluctuating necrosis marker concentrations [43], initial cardiac outputs and pump functions of slaughterhouse-based hearts.

Hypertrophic hearts are known to be more vulnerable to ischemia and reperfusion injury [44] due to dilated epicardial coronaries, reduced capillary density and vascular dilatation reserve, which reduces the diffusion of nutrients and oxygen [45] and could potentially negatively influence the cardiac arrest. The presence of dilated, hyperemic coronaries in beating pig hearts revived in the PhysioHeart™ platform has been recently confirmed by Schampaert, van ‘t Veer [21] who associated the hyperemia to an endothelial response to the organ harvest and preparation. However, whether the hyperemic circulation is related to these preparation processes or a hypertrophy-related impairment to pharmacological and physiological stimulation, as other works suggest [46, 47], is still not fully understood.

The acidic environment during cardiac storage reduced the pH of the blood pool after cardiac resuscitation. The pH balancing with sodium bicarbonate led to an increase of sodium and reduction of ionized calcium [48] which was then counter balanced with calcium chloride administration resulting in constant rise of sodium and chloride in the blood. Besides these processes, the revived hearts showed a physiological aerobic cardiac metabolism, supported by free fatty acid uptake and lactate as well as glucose metabolism similar to previous reports [40, 49–51]. The constant rise of ammonia also confirms an amino acid catabolism.

However, as the cardiac hemoperfusion progresses, essential cardiac nutrients like free fatty acids decrease and toxic waste products like ammonia increase; this is known to cause edema and to disturb oxidative phosphorylation in the mitochondria [52]. This could explain the increasing lactate values and gain of heart weight of more than 20% at the end of the experiments.

The rise of plasma free hemoglobin in our study was not significant. However, in only one experiment free hemoglobin passed 0.08 mmol/L, which occurred already from the beginning of the experiment. That could have resulted from pre-experimental blood handling. We identified the centrifugal pump as the source with the highest risk to induce hemolysis. Finally, the static concentrations of albumin, triglycerides, urea, creatinine, calcitriol but also potassium exclude the possibility that the rise of electrolytes could arise from evaporation of free water in our system.

### Epicardial electrical activity during the working mode

Electrical measurements showed physiological electrical activities of hearts revived in the PhysioHeart™ platform and during the working mode. This can be appreciated in the activation patterns presented in Fig. 10 and Fig. 11, which show unaltered electrical conduction pathways with no observable conduction block or ischemic effects in the areas of interest. Also, WPV restitution (i.e. a decrease in wave propagation velocity as the pacing frequency is increased) was observed and presented in Table 2. The analysis of restitution effects is central in the early detection of arrhythmia and in testing anti-arrhythmic drugs and devices; consequently, observing restitution effects in the PhysioHeart™ platform enables its use to investigate these phenomena within the scope of normothermic perfusion.

Although normal physiological behavior was observed during the working mode, all PhysioHeart™ experiments showed abnormally high sodium, potassium and ionized calcium concentrations in blood. These concentrations increased as the experiment progressed, this is evident from Fig. 6. The abnormally high sodium concentration translated, as observed in Table 2 and as supported by previous research [53], in high wave propagation velocity. Abnormally fast depolarization waves could induce arrhythmias because they may cause re-entrant waves or conduction block. High ionized calcium concentration in blood has also been shown to be related to longer action potentials [54] and abnormal membrane excitability [53]. Also, the observed hyperkalemia is known to cause elevated resting membrane potentials and reduced cellular excitability [55] and, consequently, arrhythmia such as atrial fibrillation or ventricular tachycardia. The use of insulin in our experiments may have support these effects as insulin leads to a dose-dependent influx of potassium into the cells [56]. This last fact was also evident because, in some experiments, stimulation protocols induced arrhythmias when pacing higher than sinus rhythm.

These observations put in evidence the importance of, simultaneously, monitoring the ion concentrations in blood and the electrophysiological activity of the cardiac tissue. In particular, the use of electrode grids within normothermic perfusion platforms could enable the detection of localized ischemia and abnormal conduction patterns that could result in arrhythmia during transport. Moreover, the monitoring of the ion concentrations in blood would also enable to determine the causes of any unphysiological electrical behavior, which can result in fast action to prevent the decreased performance of the heart.

### Achieving normal cardiac physiology during ex-vivo slaughterhouse heart perfusion

The PhysioHeart™ platform, with its starling resistor as preload and a standard four-element Windkessel model as afterload, generates flow patterns and pressure curves in the revived slaughterhouse hearts that are similar to those measured in humans [15]. For an average of 3 h, physiological and morphological cardiac characteristics, with normal electrical and metabolic activities, can be obtained without any corrective measures. Although not all blood values are physiological prior and during reperfusion, isolated beating slaughterhouse porcine hearts seem to tolerate these pathologies for a limited period. Therefore, it is inferred that the isolated working normothermic heart can be used as a baseline model to study cardiac intervention methods (LVADs, TAVI valve replacements, etc.).

These interventions may be unloading, moderate hypothermia, filtration of plasma for inflammatory components and metabolic waste, addition of nutrients and protective drugs. In view of ethical constraints regarding use of animals for short-term and uncertain-outcome experiments, the platform provides several benefits including availability, low cost, and no ethical objections.

In view of improved and prolonged preservation of the PhysioHeart™ model is the mitigation of the immune response of the pig blood. This can be achieved by separating lymphocytes and platelets, to obtain platelet and lymphocyte-poor blood in combination with administering inflammatory and autoimmune depressing drugs (i.e. dexamethasone, prednisone). The use of antibiotics and fungistatic medication would further serve to avoid infections. In addition, including anti-arrhythmic drugs in the platform will help mitigate the effects of high electrolyte concentrations in blood and, consequently, extend the time in the working mode.

We have identified that the PhyioHeart™ lacks hepatological and nephrological clearances and substance supplementations. In the upcoming future, the blood perfusate should be kept physiological and renewed constantly to maintain metabolomics and proteomic profiles and to remove toxins either with dialysis, new platelet-poor plasma [52] or a similar complex medium (i.e. HCO3, HEPES, inorganic salts, amino acids, carbohydrates, fatty acids, lipids, vitamins (Cernevit), trace elements, colloids and hormones for vasodilation like Milrinone). Dialysis and hemofiltration may be helpful by removing excess water, toxins and stabilizing the electrolyte and ion balance as the periods of arrhythmia observed in the PhysioHeart™ experiments were associated with high ion concentrations. Finally, these attempts are assumed to attenuate the loss of cardiac function in the platform and would lead to more standardized and improved experimentation.

### Limitations of the current PhysioHeart™ experiments

Despite low animal costs, big animal isolated heart experiments remain costly. Therefore, the scope of this study was rather exploratory in order to determine the boundary conditions and attempts needed to prolong physiological cardiac perfusion in our model.

All the hearts had different initial left ventricular function and electrical activity, which result in a ‘normal variation’ that may affect small-number experiments. Therefore, it is important to standardize and optimize the harvest and mounting procedures to avoid outlying negative performance at the start of the experiment. An additional limitation is that the blood pool volumes between experiments were not equal could have influenced blood marker concentrations; a standard initial volume is required to ensure consistency across experimental acquisitions. Additionally, the priming volume for deairing the circuit was not equal between experiments and, most likely, biased the baseline measurements; in future experiments, the priming volume will be considered when analyzing baseline values. Finally, one may consider non-invasive epicardial monitoring such as echocardiography, stress-strain imaging and speckle imaging to detect early signs of ischemia that may result in corrective or supporting actions. Moreover, the use of square grids limits the electrophysiological study to a small region of the heart; the use of more sophisticated electrode arrays, capable of making whole-heart measurements, would enable a more comprehensive electrophysiological study. Finally, micro-puncture histology during and at the end of the experiments may be useful as a hard outcome parameter.

## Conclusion

The isolated working slaughterhouse heart is a practical (e.g. abundant numbers, no need to specifically sacrificed laboratory animals, no ethical approval of study protocols, etc.) and cost-efficient model to perform investigative and therapeutic experiments. This study was meant to identify factors limiting these experiments of isolated slaughterhouse porcine heart in the PhysioHeart™ perfusion model. Our findings confirm the viability and function loss of the isolated slaughterhouse hearts are best described by the phenomenon of “time-dependent multi-organ” failure. The blood parameters, biochemical and electrophysiological changes observed in the PhysioHeart™ platform provide a better understanding of the necessary effort to overcome the challenges. Diligent and strictly protocolized harvesting and installation will reduce variation at the start of the PhysioHeart™ platform experiment. This study identified several mechanisms and provided explanations of the potential sources which limit the ex vivo cardiac viability and perfusion time which in turn could be corrected. Perfusate renewal and clearance has been recognized as crucial for prolonged cardiac perfusion in the PhysioHeart™ model which could be of potential interest for other heart platforms.

## Data Availability

The datasets used and/or analyzed during the current study are available from the corresponding author on reasonable request.

## Abbreviations

AP: Aortic pressure
ASAT: Aspartat-aminotransferase
ATP: Arterial pressure
CK: Creatine kinase
DPG: 2,3-diphosphoglycerate
FFP: Fresh frozen plasma
LDH: L-Lactatdehydrogenase
LV: Left ventricle
NI: National instruments
RVOT: Right ventricular outflow tract
UEG: Unipolar epicardial electrograms
WPV: Wave propagation velocity
